# Study on the host–guest complex of dicyclohexanocucurbit[6]uril and 2-phenylbenzimidazole, and its recognition effect toward Fe^3+^

**DOI:** 10.1098/rsos.211280

**Published:** 2021-12-22

**Authors:** Jun Zheng, Lian An, Jie Gao, Lin Zhang, Xinan Yang, Weiwei Zhao, Siyuan Cheng, Peihua Ma

**Affiliations:** Key Laboratory of Macrocyclic and Supramolecular Chemistry of Guizhou Province, Guizhou University, Guiyang 550025, People's Republic of China

**Keywords:** CyH_2_Q[6], 2-phenylbenzimidazole, host–guest interaction, ion recognition, limit of detection

## Abstract

This paper has selected dicyclohexanocucurbit[6]uril (CyH_2_Q[6]) as the host and 2-phenylbenzimidazole (**G**) as the guest to investigate the host–guest interaction mode between CyH_2_Q[6] and **G**. Under acidic conditions, the complex was characterized using nuclear magnetic resonance, ultraviolet and fluorescence spectroscopy. The results show that the molecular ratio of CyH_2_Q[6] to **G** is 2 : 1. The crystals were cultured with ZnCl_2_ as a structural inducer under acidic conditions and single crystal X-ray diffraction showed that the molecular ratio of CyH_2_Q[6] to **G** is 1 : 3. The G@CyH_2_Q[6] was used as a fluorescent probe to identify metal cations. The probe exhibits a good selective recognition effect toward Fe^3+^ ions, which involves a reduced fluorescence intensity with a limit of detection of 1.321 × 10^–6^ mol l^–1^.

## 1. Introduction

Benzimidazole compounds [1–5] are aromatic heterocyclic compounds containing two nitrogen atoms. Benzimidazole derivatives and metal complexes exhibit good biological activity. Benzimidazole and its derivatives containing an imidazole ring have important medicinal value and have important applications as anti-fungal, anti-rheumatic, deworming, analgesic, anti-inflammatory and anti-cancer agents, among others [6–13]. Fe^3+^ is widely distributed in nature and is one of the indispensable trace elements found in the human body. It plays an important role in metabolism. Cucurbit[n]uril (Q[n] or CB[n]) [14–28] is a new type of macrocyclic compound discovered after cyclodextrins, crown ethers and calixarenes. However, most ordinary cucurbit[n]uril have poor solubility, the development of cucurbit[n]urils has been significantly limited. Through the efforts of some researchers, several modified cucurbit[n]urils, such as methyl-, hydroxyl-, cyclopentyl- and cyclohexyl-substituted cucurbit[n]urils have been reported [29–35]. Dicyclohexanocucurbit[6]uril (CyH_2_Q[6]) is a modified cucurbituril, which has made great contributions in host–guest chemistry, coordination chemistry, etc. [36–39].

**Figure 1.**
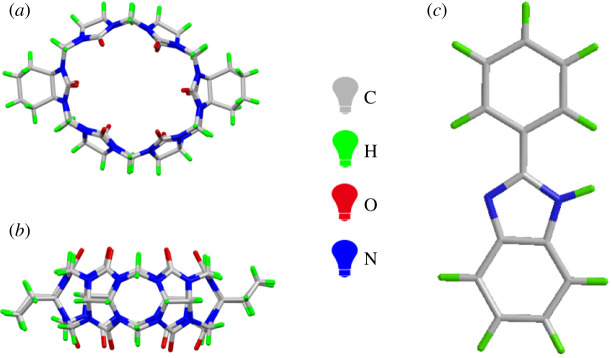
The structure of (*a*,*b*) CyH_2_Q[6] and (*c*) 2-phenylbenzimidazole.

Herein, CyH_2_Q[6] was selected as the host and 2-phenylbenzimidazole (**G**) used as the guest to study the host–guest interaction (figure 1). Nuclear magnetic resonance (NMR), ultraviolet (UV) and fluorescence spectroscopy, and single crystal X-ray diffraction were used to characterize the host–guest complex. Regarding the study of cucurbit[nuril induced to sensing for metal ions many scientific researchers have made great efforts and have reported in related fields^40^,^41^]. Specifically, CyH_2_Q[6] with 2-phenylbenzimidazole was designed as a fluorescent probe and its ability to recognize metal ions was explored using the change in its fluorescence intensity. The results show that the fluorescent probe can selectively recognize Fe^3+^ cations, which exhibit a reduced fluorescence intensity and can be used to detect the Fe^3+^ concentration in a sample using the reduced fluorescence effect.

## 2. Experimental section

### 2.1. Instruments and reagents

Bruker D8 Venture X-ray single crystal diffractometer (Bruker, Germany), JNM-ECZ400S/L1 400M superconducting NMR spectrometer, a UV-2700 dual-beam UV–vis spectrophotometer (Shimadzu Instruments Co. Ltd), VARIANCARYE-CLIPSE fluorescence spectrophotometer (Varian, USA). All materials were reagent grade and used without any further purification. CyH_2_Q[6] (purity ≥97%) was prepared in the Key Laboratory of Macrocyclic and Supramolecular Chemistry of Guizhou Province, China.

### 2.2. Interaction between CyH_2_Q[6] and 2-phenylbenzimidazole: ^1^H NMR titration

CyH_2_Q[6] was added to deuterated water containing a certain amount of deuterated hydrochloric acid to prepare a solution with a concentration of 1.0 × 10^–3^ mol l^–1^ (pH = 1). Fifty microlitres of this solution and 450 µl of deuterium chloride solution was loaded into an ^1^H NMR tube and a certain amount of 2-phenylbenzimidazole (n(**G**)/n(CyH_2_Q[6]) = 0.2, 0.4, 0.6, …) was added in sequence and the ^1^HNMR spectrum recorded at 293 K.

### 2.3. UV and fluorescence titration

CyH_2_Q[6] and 2-phenylbenzimidazole were formulated into an acidic aqueous solution (pH = 1) with a concentration of 1.00 × 10^–2^ and 1.00 × 10^–3^ mol l^–1^, respectively, to keep the concentration of the guest unchanged and an appropriate amount of CyH_2_Q[6] (n(G)/n(CyH_2_Q[6]) = 0.2, 0.4, 0.6, …) was then added and analysed using UV absorption and fluorescence spectroscopy.

### 2.4. Crystal culture and testing

CyH_2_Q[6] (10 mg, 9.05 µmol) and 2-phenylbenzimidazole (5 mg, 25.76 µmol) were dissolved in a 10 ml round bottom flask containing 5 ml of HCl (3 mol l^−1^), and ZnCl_2_ (0.67 g) was added. The resulting mixture was shaken for 3 min at room temperature and heated to reflux for 5 min with stirring. The mixture was cooled and left to stand for about one week to obtain colourless crystals. A regular and transparent crystal of the complex was selected and fixed on a glass filament using petroleum jelly for X-ray single crystal diffraction analysis on a Bruker D8 Venture X-ray single crystal diffractometer in ω-scan mode using graphite to monochromatize the Mo-K*α* rays (*λ* = 0.71073 Å, *μ* = 0.828 mm^–1^). The crystal data were collected and Lorentz polarization, and absorption correction carried out. SHELXT-14 and SHELXL-14 program packages were used for structural analysis and full matrix least-squares refinement. All non-hydrogen atoms were anisotropically refined using the analytical expression of the neutral atom scattering factor and combined with anomalous dispersion correction. The SQUEEZE program in the PLATON package was used to delete some of the disordered solvent molecules. The X-ray crystallographic data for structures reported in this study have been deposited in the Cambridge Crystallographic Data Center under accession no. CCDC: 2100651. These data can be obtained free of charge via https://www.ccdc.cam.ac.uk/data_request/cif. The crystal data and structure modification parameters are shown in table 1.

**Table 1.** The crystallographic parameters of the complex.

empirical formula	C_83_H_82_Cl_8_N_30_O_14_Zn_2_	*D*c (g cm ^−3^)	1.349
*M*_r_	2138.12	F(000)	4392
crystal system	monoclinic	*μ* (mm^−1^)	0.729
space group	*P*2_1_/c	data/params	17 898/1187
*a* (Å)	15.6869(8)	*R*_int_	0.1002
*b* (Å)	48.224(2)	*R*[I > 2*σ*(I)]^a^	0.1320
*c* (Å)	14.3174(6)	*wR*[I > 2*σ*(I)]^b^	0.3543
*α* (deg)	90	*R*(all data)	0.1779
*β* (deg)	103.561(2)	*wR*(all data)	0.3808
*γ* (deg)	90	GOF (F^2^)	1.350
*V* [Å^3^]	10529.0(9)	*T* (K)	300.0
Z	4	CCDC	2 100 651

^a^Conventional *R* on *Fhkl*: ∑||*F_o_*| − |*F_c_*||/∑|*F_o_*|.

^b^Weighted R on |*Fhkl*|^2^: $\sum [w{\left(F_{o}^{2}-F_{c}^{2}\right)}^{2}]/\sum {\left[w{\left(F_{o}^{2}\right)}^{2}\right]}^{1/2}$.

### 2.5. Selective fluorescence measurement of metal ions

First, all the metal ions studied were accurately prepared into 0.2 mol l^–1^ solutions using deionized water and then, under acidic conditions (pH = 1), a solution of **G** at a concentration of 5 × 10^−5^ mol l^–1^ prepared with deionized water was used to form a **G**@CyH_2_Q[6] solution. Three millilitres of the 5 × 10^–5^ mol l^–1^ **G**@CyH_2_Q[6] solution was added to a quartz fluorescent cuvette and the different metal cation solutions added. The amount of metal cation added to the **G**@CyH_2_Q[6] probe was 10 times the equimolar concentration. A fluorophotometer was used to detect the fluorescence intensity change at the maximum excitation wavelength (*λ*_ex_ = 298 nm); the slit width was 5 nm/5 nm, voltage was 400 V, and scanning range was 250–600 nm.

### 2.6. Fluorescent quenching experiment of metal ions

Under acidic conditions (pH = 1), a solution of **G**@CyH_2_Q[6] and Fe^3+^ at a concentration of 5 × 10^–5^ mol l^–1^ was prepared with deionized water and 3 ml of the resulting solution was added to a quartz fluorescent cuvette. Different metal cation solutions were added to the solution; the amount of metal cation added was 10 times the equimolar concentration of the **G**@CyH_2_Q[6] and Fe^3+^ solution. After mixing uniformly, a fluorophotometer was used to detect the fluorescence signal intensity within 2 min at the maximum excitation wavelength (*λ*_ex_ = 298 nm); the slit width was 5 nm/5 nm, voltage was 400 V, and scanning range was 250–600 nm.

### 2.7. Titration experiment of Fe^3+^

Three millilitres of a 5 × 10^–5^ mol l^–1^ solution of the **G**@CyH_2_Q[6] probe was added to a quartz cuvette, followed by Fe^3+^ and **G**@CyH_2_Q[6] = 0, 0.5, 1.0, 1.5, 2.0, 2.5. After adding 3.0, 3.5, 4.0, … , 28.0 equivalent of the Fe^3+^ solution to the quartz cuvette in turn and mixing uniformly, a fluorophotometer was used to detect the fluorescence signal intensity within 2 min at the maximum excitation wavelength (*λ*_ex_ = 298 nm); the slit width was 5 nm/5 nm, voltage was 400 V, and scanning range was 250–600 nm.

## 3. Results and discussion

### 3.1. ^1^HNMR Titration of the host–guest interaction

**Figure 2.**
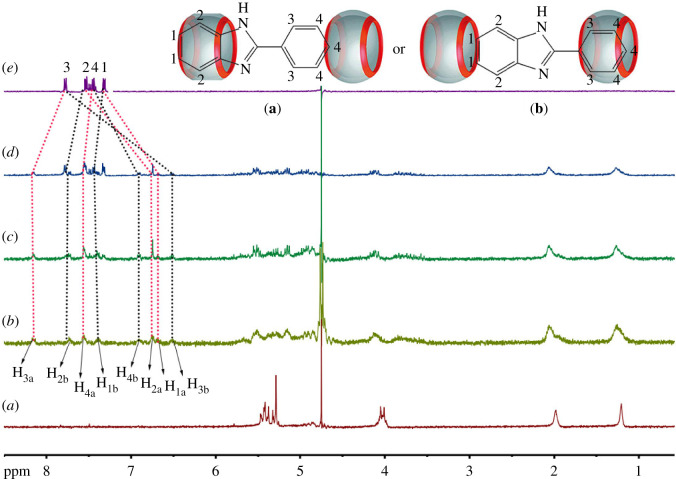
^1^HNMR spectra (25°C, 400 MHz) of the interaction between CyH_2_Q[6] and **G** recorded in D_2_O upon adding (*a*) 0, (*b*) 0.4, (*c*) 0.6 and (*d*) 1.0 equivalents of **G**, and (*e*) pure **G**.

Figure 2 shows the ^1^HNMR titration spectra obtained when adding different equivalents of 2-phenylbenzimidazole (**G**) to CyH_2_Q[6]. It can be seen from the figure that, upon the addition of **G**, each proton peak of **G** splits into two groups of peaks, moving to the high field and low field, respectively. A preliminary judgement can be made on this. There are two modes of action for **G** and CyH_2_Q[6] (shown by the black and red curves in figure 2), namely mode **a** and mode **b**. In mode **a**, the benzimidazole part enters the cavity of CyH_2_Q[6] and the benzene ring is outside the port of CyH_2_Q[6]; the benzimidazole part of **G** is shielded and the benzene ring is unshielded. In mode **b**, the benzene ring enters the cavity of CyH_2_Q[6] and the benzimidazole part is outside the port of CyH_2_Q[6]; the benzene ring part of G is shielded and the benzimidazole is unshielded. When 0.4 equivalents of **G** were added, the chemical shift values of H2a and H1a in **G** correspond to their free state moving 0.74 and 0.64 ppm to the high field, respectively; H3a and H4a move 0.37 and 0.09 ppm to the low field, respectively. The chemical shift values of H3b and H4b correspond to their free state moving 1.27 and 0.52 ppm to the high field, respectively; H2b and H1b move 0.24 and 0.07 ppm to the low field, respectively. When 0.6 equivalents of **G** were added, the chemical shift of the proton signal peaks of **G** are the same as the chemical shifts observed after adding 1.0 equivalent, and a free guest peak appears at the same time. This shows that the amount of the guest was excessive, indicating that the ratio of CyH_2_Q[6] to **G** was 2 : 1.

### 3.2. UV absorption spectroscopy of the host–guest interaction

**Figure 3.**
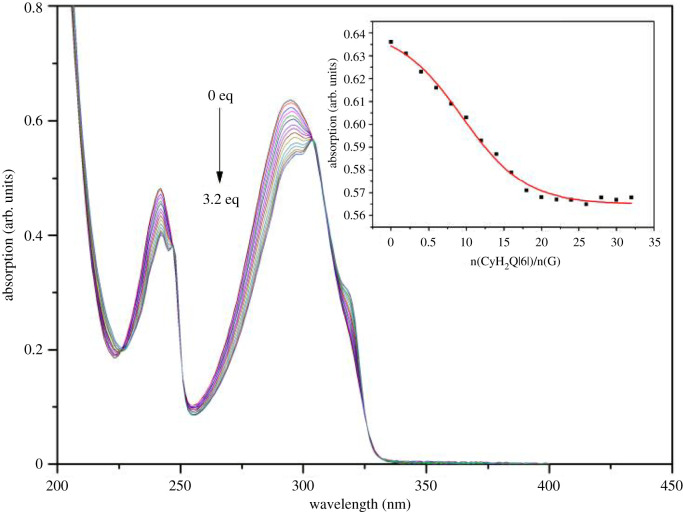
UV spectra and trend chart obtained for the solution of **G** (5 × 10^–5^ mol l^–1^) upon gradually adding CyH_2_Q[6] (0, 0.2, 0.4, 1.6, … , 3.2 equivalents).

Figure 3 shows the UV absorption spectra obtained upon the interaction of CyH_2_Q[6] with **G**. n(CyH_2_Q[6])/n(G) = 0.2, 0.4, 0.6, … , 3.2, was added to the guest solution and the UV spectra recorded. As the amount of CyH_2_Q[6] added to the guest solution increases, the UV absorption of the guest shows a regular change and gradually decreases. When the host–guest ratio was 2 : 1, the fitting curve exhibits an inflection point and as the concentration of CyH_2_Q[6] further increases, the absorption intensity basically remains unchanged, indicating that the ratio of CyH_2_Q[6] to **G** was 2 : 1.

### 3.3. Crystal structure

**Figure 4.**
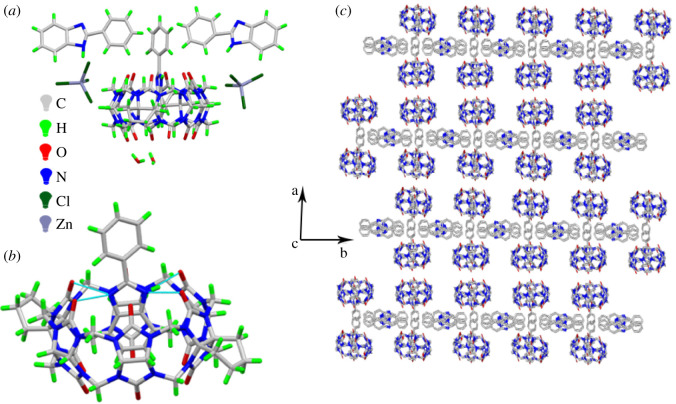
Crystal structure of CyH_2_Q[6] and **G**: (*a*) Asymmetric structural unit, (*b*) host–guest interaction, and (*c*) stacked diagram of the structure along the c-axis.

CyH_2_Q[6] and 2-phenylbenzimidazole (**G**) were added with ZnCl_2_ as a structural inducer and the crystal structure of the resulting complex was determined using X-single crystal diffraction. The asymmetric structural unit of the complex includes one CyH_2_Q[6] molecule, three molecules of **G**, two [ZnCl_4_]^2–^ ions and two free H_2_O molecules (figure 4*a*). Figure 4*b* clearly shows that **G** enters the cavity of the cucurbit[n]uril, and the two nitrogen atoms on the imidazole ring interact with the carbonyl oxygen atoms at the port of the cucurbit[n]uril via hydrogen bonding (as shown in figure 4*b*). Figure 4*c* shows the stacking diagram of the crystal structure of the complex viewed along the c-axis. The other two molecules of 2-phenylbenzimidazole act as a bridge connecting the two layers of cucurbit[n]uril, so that the complexes form a regular arrangement.

### 3.4. Fluorescence spectroscopy of the host–guest interaction

**Figure 5.**
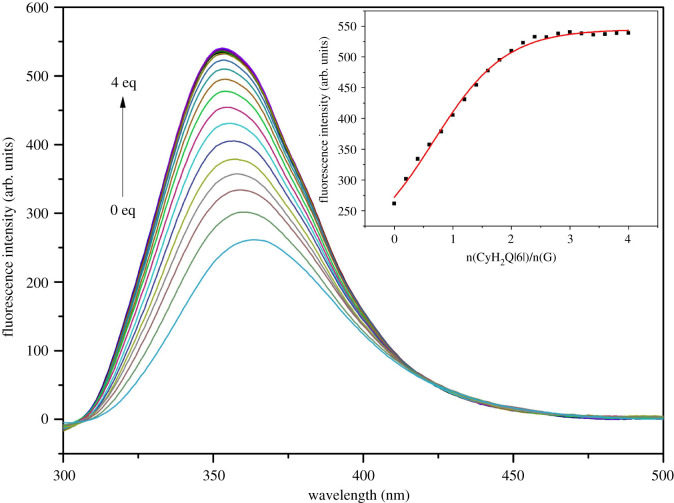
Fluorescence titration spectra of the host–guest interaction between CyH_2_Q[6] and **G**.

Figure 5 shows the fluorescence spectra obtained for the interaction between CyH_2_Q[6] and **G** at the maximum fluorescence emission wavelength of 363.07 nm and maximum absorption wavelength of 295 nm. Three millilitres of a 5 × 10^–5^ mol l^–1^ solution of **G** was added to a quartz cuvette and CyH_2_Q[6] slowly added to the guest solution at the following ratios: (CyH_2_Q[6])/n(G) = 0.2, 0.4, 0.6, … , 4.0. The UV spectra indicate that the fluorescence intensity gradually increases, but upon reaching n(CyH_2_Q[6])/n(G) = 2 : 1, the fluorescence intensity only gradually changes.

### 3.5. Determination of the metal cations selectivity using fluorescence spectroscopy

**Figure 6.**
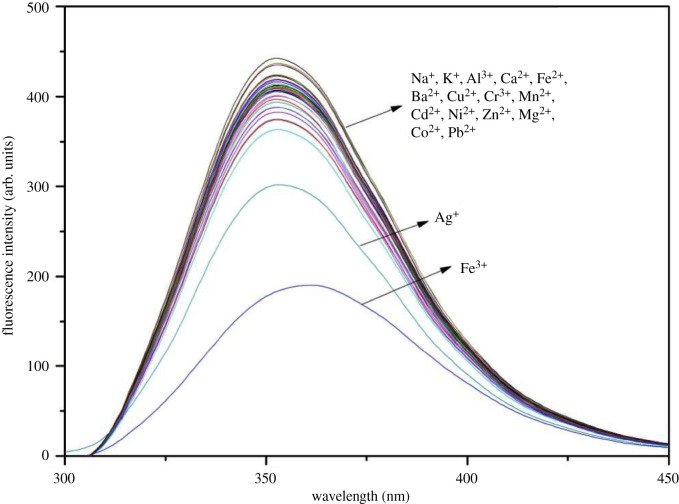
The fluorescence spectra of the **G**@CyH_2_Q[6] (5 × 10^–5^ mol l^–1^) probe in the presence of various metal cations (10 eq) recorded in an acidic aqueous solution (pH = 1).

The selectivity of **G**@CyH_2_Q[6] toward common metal cations was monitored using fluorescence spectroscopy (figure 6). In the fluorescence range of 600 nm, the **G**@CyH_2_Q[6] probe exhibits a strong fluorescence intensity. Upon adding ten equivalents of different metal cations (Hg^2+^, Ca^2+^, Na^+^, Mg^2+^, Al^3+^, Cd^2+^, Cu^2+^, Pb^2+^, Ni^2+^, Co^2+^, Cr^3+^, …), most of the metal cations do not affect the fluorescence intensity of the host and guest complex. However, Fe^3+^ ions cause a significant decrease in the fluorescence intensity, which indicates that the probe was highly selective toward Fe^3+^ ions.

### 3.6. Investigation of the interference of metal cations on the probe using florescence spectroscopy

**Figure 7.**
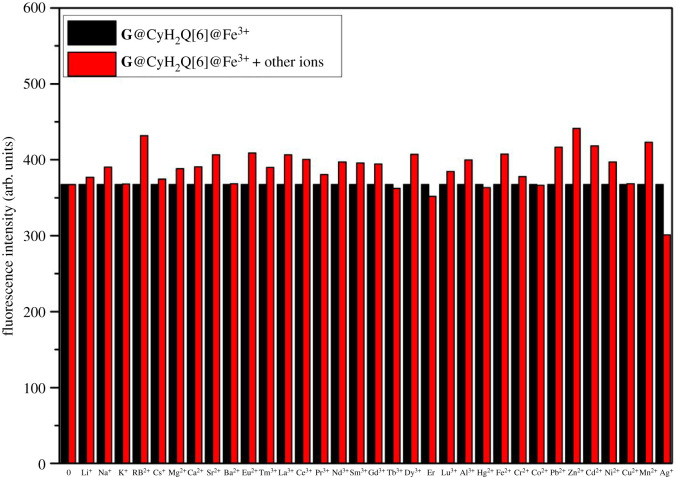
The change in the fluorescence intensity after adding different metal ions to the **G**@CyH_2_Q[6] and Fe^3+^ system.

Figure 7 shows the interference of other metal cations on the probe's recognition of Fe^3+^. The specific operation of this experiment was as follows: 3 ml of a 5 × 10^–5^ mol l^–1^ solution of **G**@CyH_2_Q[6] and Fe^3+^ was added to a quartz cuvette and its fluorescence intensity measured. Different metal cation solutions (the concentration of the metal cations was 10 times that of the probe) were then added to compare whether the fluorescence intensity changes upon adding the different metal cations. The black bars in the figure shows the fluorescence intensity of the **G**@CyH_2_Q[6] and Fe^3+^ solution at an excitation wavelength of 298 nm and the red bars indicate the fluorescence intensity of the **G**@CyH_2_Q[6] and Fe^3+^ solution upon the addition of other metal cations. It can be seen from the figure that after adding other metal cations to the **G**@CyH_2_Q[6] and Fe^3+^ solution, the fluorescence intensity was changed, but the degree of change was not large. This fully shows that the Ag^+^, Tb^3+^, Er^3+^ and Hg^2+^ ions have weak interference to the recognition of Fe^3+^, and other metal cations cannot interfere with the recognition of Fe^3+^ by the complex.

### 3.7. Limit of detection for the Fe^3+^ titration detection probe

**Figure 8.**
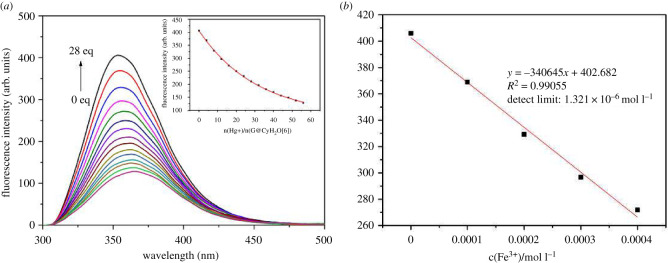
Fluorescence titration of Fe^3+^ and **G**@CyH_2_Q[6] (*a*) and fluorescence intensity calibration curve (*b*).

As shown in figure 8, the concentration of the fixed probe **G**@CyH_2_Q[6] is 5 × 10^–5^ mol l^–1^, and the Fe^3+^ solution of 0.2 molar ratio is added dropwise in turn. With the increase of the concentration of Fe^3+^ added, the fluorescence intensity gradually decreases. According to the detection limit formula (3*σ*/K), the detection limit of **G**@CyH_2_Q[6] for Fe^3+^ is calculated, and the detection limit is 1.321 × 10^–6^ mol l^−1^, and the linear correlation is *R*^2^ = 0.99055. It is lower than the maximum Fe^3+^ content of 0.3 mg l^−1^ specified in the national drinking water sanitation standard (GB5749-2006), so the probe **G**@CyH_2_Q[6] can effectively identify Fe^3+^ in drinking water.

## 4. Conclusion

In this paper, 2-phenylbenzimidazole was selected as the guest molecule and CyH_2_Q[6] was used as the host to form an inclusion complex, which was used as a fluorescent probe to identify metal cations. Firstly, the interaction between the CyH_2_Q[6] host and 2-phenylbenzimidazole guest in the liquid phase was studied using NMR, UV and fluorescence spectroscopy. The experimental results showed that CyH_2_Q[6] and 2-phenylbenzimidazole formed a 2 : 1 inclusion compound. There are two modes of action under acidic conditions (pH = 1). One mode is the entry of the benzimidazole into the cavity of the cucurbit[n]uril and the portal interacts with the benzene ring. The other mode is that the benzene ring enters into the cavity of the cucurbit[n]uril and the benzimidazole interacts with the portal. The interaction between CyH_2_Q[6] and 2-phenylbenzimidazole in the solid phase was studied using single crystal X-ray diffraction. The crystal structure results showed that CyH_2_Q[6] forms a 1 : 3 inclusion compound with 2-phenylbenzimidazole. The recognition of metal cations between CyH_2_Q[6] and 2-phenylbenzimidazole was studied and the fluorescent probe (**G**@CyH_2_Q[6]) was constructed under acidic conditions (pH = 1). The recognition ability of the probe toward metal cations has been studied. The **G**@CyH_2_Q[6] probe can selectively identify Fe^3+^ ions with a limit of detection of 1.321 × 10^–6^ mol l^–1^ and has a strong ability to resist cation interference.

## Data accessibility

Data available from: https://www.ccdc.cam.ac.uk/data_request/cif.
